# Efficient Structure Resonance Energy Transfer from Microwaves to Confined Acoustic Vibrations in Viruses

**DOI:** 10.1038/srep18030

**Published:** 2015-12-09

**Authors:** Szu-Chi Yang, Huan-Chun Lin, Tzu-Ming Liu, Jen-Tang Lu, Wan-Ting Hung, Yu-Ru Huang, Yi-Chun Tsai, Chuan-Liang Kao, Shih-Yuan Chen, Chi-Kuang Sun

**Affiliations:** 1 Department of Electrical Engineering and Graduate Institute of Photonics and Optoelectronics, National Taiwan University, Taipei 10617, Taiwan; 2 Department of Clinical Laboratory Sciences and Medical Biotechnology, College of Medicine, National Taiwan University, Taipei 10617, Taiwan; 3 Institute of Biomedical Engineering, National Taiwan University, Taipei 10617, Taiwan; 4 Department of Electrical Engineering and Graduate Institute of Communication Engineering, National Taiwan University, Taipei 10617, Taiwan; 5 Molecular Imaging Center and Graduate Institute of Biomedical Electronics and Bioinformatics, National Taiwan University, Taipei, 10617, Taiwan

## Abstract

Virus is known to resonate in the confined-acoustic dipolar mode with microwave of the same frequency. However this effect was not considered in previous virus-microwave interaction studies and microwave-based virus epidemic prevention. Here we show that this structure-resonant energy transfer effect from microwaves to virus can be efficient enough so that airborne virus was inactivated with reasonable microwave power density safe for the open public. We demonstrate this effect by measuring the residual viral infectivity of influenza A virus after illuminating microwaves with different frequencies and powers. We also established a theoretical model to estimate the microwaves power threshold for virus inactivation and good agreement with experiments was obtained. Such structure-resonant energy transfer induced inactivation is mainly through physically fracturing the virus structure, which was confirmed by real-time reverse transcription polymerase chain reaction. These results provide a pathway toward establishing a new epidemic prevention strategy in open public for airborne virus.

In the past few decades, tremendous efforts have been made to kill airborne viruses such as severe acute respiratory syndrome (SARS) coronavirus or influenza viruses, which have caused catastrophic illness worldwide. Current airborne virus epidemic prevention to be used in public space includes strong chemical inactivation, UV irradiation, and microwave thermal heating. All these methods affect the open public. In 1980s, Robach *et al*.^1^ and Cerf 2 demonstrated that ultrasonic energy can be absorbed by viruses. In 2000, Babincová *et al*.^3^ hypothesized that viruses can be inactivated by generating the corresponding resonance ultrasound vibrations of viruses, which is in the GHz region. Based on this hypothesis, several groups started investigating the vibrational modes of viruses in this frequency range^4^,^5^,^6^,^7^. Recently we demonstrated that dipolar mode of the confined acoustic vibrations (CAVs) inside viruses can be resonantly excited by microwaves of the same frequency with a resonant microwave absorption effect^8^. The observed microwave resonance absorption phenomenon indicates a possible structure-resonant energy transfer (SRET) effect from electromagnetic waves (EM waves) to CAVs of viruses. Theoretically this SRET process is an efficient way to excite the vibrational mode of the whole virus structure due to a 100% energy conversion of a photon into a phonon of the same frequency, but the overall SRET efficiency is also related to the mechanical properties of the surrounding environment^9^, which influences the quality factor of the oscillator (virus). A study on the SRET efficiency to inactive virus is thus highly desired and it will determine if this SRET phenomenon provides a solution to inactivate airborne viruses in open public for epidemic prevention.

In this article, we show that SRET from microwave to virus can be efficient enough so that airborne virus was inactivated with reasonable microwave power density safe for the open public. To investigate the SRET efficiency from EM waves to CAVs in viruses, we first developed a theoretical model to describe the relation between the induced stress and the field magnitude of the illuminating microwave. Since the viruses could be inactivated when the induced stress fractures the structure of viruses, we propose to explore the SRET efficiency from microwaves to viruses through measuring the virus inactivation threshold. Based on the proposed model, we studied the inactivation ratio of influenza A (H3N2) virus at dipolar-mode-resonance and off-resonance microwave frequencies as well as with different microwave powers. Plaque assay^10^ was then applied to calculate the titer of virus samples before and after the microwave illumination. Our results indicate efficient SRET from microwave to viruses, which resulted in higher inactivation ratio of viruses at the dipolar resonant frequency. At the resonant frequency, the microwave power density threshold for H3N2 inactivation was found to be below the IEEE safety standard, also agreeing well with our developed theoretical model. The real-time reverse transcription polymerase chain reaction (real-time RT-PCR) method^11^ was further performed to confirm that the main inactivation mechanism is through physically fracturing the viruses while the RNA genome was not degraded by the microwave illumination, supporting the fact that our studied SRET mechanism is fundamentally different from the microwave thermal heating effect. These results provide a pathway toward establishing a new epidemic prevention strategy in open public for airborne virus.

## Modelling

From the transmission electron microscope images, people knew that the virions of influenza viruses are basically spherical balls packing genomes inside. Since the protein and genome have similar mechanical properties^8^, for the estimation of dipolar vibration frequencies, we treat the virion as a homogenous sphere.

### Dipolar Mode of a Homogeneous Sphere

Due to the spatial confinement, not only electronic but also acoustic energy quantization has been observed in low dimensional systems such as quantum dots and nano-wires. In 1882, Lamb studied the torsional (TOR) and spheroidal (SPH) modes of a homogeneous free sphere by considering the stress-free boundary condition on the surface^12^. Among these modes, the SPH mode with 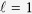 allows dipolar coupling^13^ and the corresponding eigenvalue equation can be expressed as^14^,^15^:


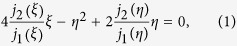


where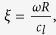 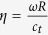 , 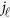 is the spherical Bessel function of the first kind, **ω** is the angular frequency of the vibrational mode, *R* is the radius of the nano-sphere, *c*_*l*_and *c*_*t*_are longitudinal and transverse sound velocities respectively. A comparison between the commonly observed 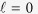
breathing mode and the 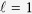 dipolar mode can be found in Supplementary online. the Since the 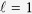 dipolar mode of a nano-sphere cannot be detected by the light scattering experiments^16^, it was not observed until a previous study of the resonant excitation of dipolar mode through THz wave or microwave excitations^17^,^18^ when the core and shell of the nano-sphere have permanent charge separation. Once the resonantly oscillating electric field was applied to the nano-sphere, opposite displacement between core and shell was generated, thus excited the dipolar mode vibrations. Compared with the breathing (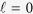) and quadrapolar (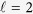) modes, dipolar mode (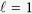) is the only SPH mode to directly interact with the EM waves whose wavelength is much longer than the particle’s size. Due to the permanent charge separation nature of viruses, in 2009, dipolar coupling with 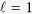 CAVs is confirmed to be the mechanisms responsible for microwave resonant absorption in viruses by treating spherical viruses as free homogeneous nanoparticles^8^,^9^.

**Figure 1:**
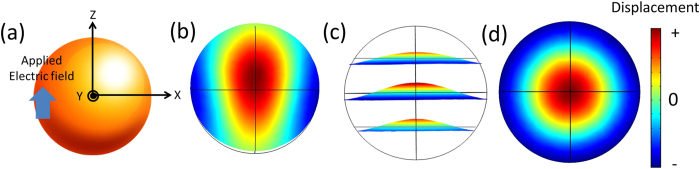
(**a**) Schematic showing a homogeneous sphere and applied electric field (**b**) Displacement field distribution of the x-z plane (y = 0) of the sphere, (**c**) side view of the distortion of the x-y plane at different z location and (**d**) top view of the displacement field distribution of the equator plane (z = 0) of the sphere when dipolar resonance mode is excited.

Figure 1 shows the simulated displacement field of the dipolar mode (calculated by the finite element method, COMSOL Multiphysics, COMSOL, Inc.) of a homogeneous sphere (mass density and viscoelastic properties are constant throughout the sphere). We define the relative displacement direction of the dipolar mode as the z-direction, which will also be the field direction of the applied EM waves discussed in the next section. By plotting the displacement field of the x-z plane (y = 0) of the sphere, the opposite displacement between the core and shell regions can be clearly observed in Fig. 1(b). Meanwhile Fig. 1 (c) shows the side view of the distortion of the x-y plane of the sphere at different z locations, which concludes that the maximum distortion occurs on the equatorial plane (z = 0) of the sphere. Figure 1(d) shows the top view of the displacement field of the equatorial plane (z = 0). It is interesting to find out that the magnitude of averaged positive displacement (inner region) is 1.27 times the magnitude of the averaged negative displacement (outer region), while positive and negative displacements occupy 42% and 58% area, respectively. Furthermore one can find that the maximum magnitude of the displacement, occurring either at the very center or the outer surface of the equatorial plane, is approximately twice of the averaged magnitude of the displacement.

### A Damped Mass-Spring Model

In this work, microwaves were applied to excite the dipolar resonance of the whole virus structure. By exciting the dipolar mode of the nanosphere, core and shell with opposite charge distributions would move in opposite directions and will resonate like a damped mass-spring system^17^. Our following analysis is similar to the Drude-Lorentz model describing the light-atom interaction, which connects a damped mass-spring system to the quantum-mechanical electronic resonant transitions. In the damped mass-spring system by adopting the reduced mass (m*) of core and shell in the analysis, the relative motion of the displacement can be shown in the following equation:


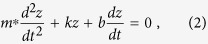


where *z* is the relative displacement between the core and shell; *b* is the damping coefficient, which is related to the surrounding environment; *k* is the effective spring constant of this system. By assuming *z(t)* proportional to *exp(iωt)*, one can solve the complex angular frequency of the resonator as:


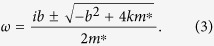


Therefore the decay rate of the oscillation equals to the imaginary part of the frequency (b/2m*), which corresponds to *ω*_*0*_*/2Q*^19^:


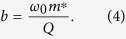


The intrinsic resonance angular frequency (*ω*_*0*_) of this system is (*k/*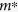)^0.5^. *Q* is the quality factor of the resonator. From equation (4), stronger damping increased the energy transfer between the resonator and its surrounding environment, which decreases the confinement of the vibration and leads the lower *Q*. Now we approximate that a spherical virus is like a homogeneous sphere but with opposite and equal charges in the core and shell regions. When the oscillating electric field (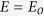
*cos*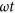) of microwaves is applied to the system, forced displacements would be induced with the same frequency as the applied microwaves. The equation of motion now needs to include the force induced by the applied electric field (*qE*), where *q* is the total amount of charge distributed in the core and shell region of a virus:


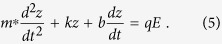


We describe the forced displacement as 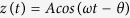, where *A* is the amplitude of the forced displacement and 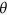 is the phase delay between the displacement and the applied electric field. By solving the particular solution of this differential equation, one can obtain the phase delay and the amplitude of the forced oscillating displacement as


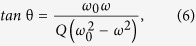


and


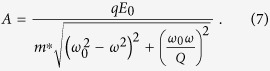


The instantaneous power absorption of this system is then described as the following equation, where *v* is the velocity of the oscillating motion^17^:


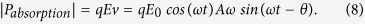


By integrating over one full cycle, one can obtain the average power absorption from the system:


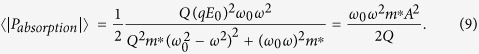


Then the absorption cross-section 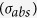 of the virus can be obtained by setting the input power flux as 
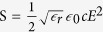
 with^20^


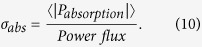


where 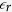 is the relative permittivity in the system and *c* is the speed of light in vacuum.

### Threshold to Fracture a Virus

With oscillating dipolar vibration to inactivate a virus, the most possible mechanism is to fracture the most outer surface of the equatorial plane (z = 0) due to the location of the maximum distortions, as illustrated in Fig. 1 (c,d). For influenza viruses, this corresponds to the lipid membrane of the envelope. To estimate the maximum induced stress 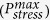 on the equatorial plane, we divide the maximum induced force by the area of the shell region (defined by the moving direction in the approximate model) on the equatorial plane. Following above discussion, we found that the maximum induced stress is twice the average value and the shell region covers 58% of the equatorial plane:


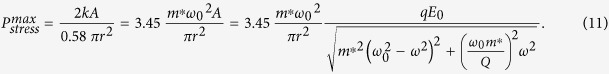


If the required stress threshold 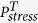 to fracture a virus can be obtained, the threshold electric field magnitude 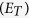 of the incident microwaves can also be obtained by using:


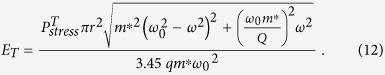


**Figure 2:**
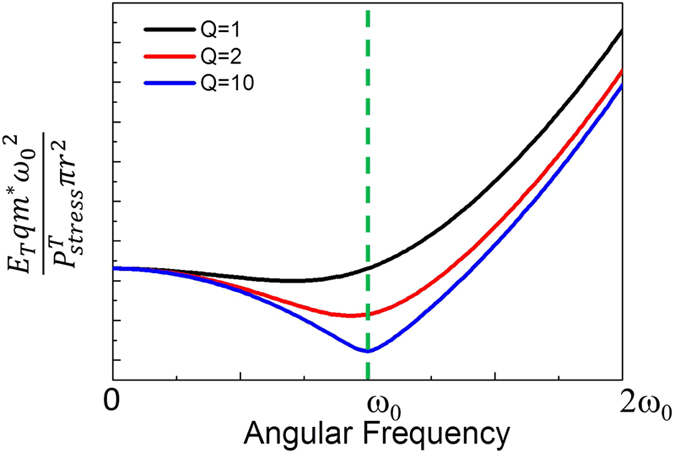
Threshold electric field magnitudes of the incident EM waves to fracture a virus as a function of angular frequency with different *Q*.

Figure 2 shows the threshold magnitude of the incident electric field at different frequencies with different *Q* based on equation (12) with a fixed 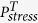 threshold value. One can observe that the minimum of the threshold electric field magnitude occurs when the applied frequency is closed to the intrinsic resonant frequency. Moreover cavity quality factor *Q* plays a major role. By changing the pH value of the solution, charge status of viral surface can be modulated, which affects the *Q* of the vibration. For example, previous studies indicated that the cavity *Q* of spherical viruses ranges between 2–10 by raising the pH value of the solution from 5.4 to 7.4^8^. With increased *Q*, more energy can be confined inside the resonator, which leads to much lower microwave field threshold magnitude at the resonant frequency.

To experimentally study the efficiency of the SRET from microwaves to CAVs of spherical viruses, influenza A virus subtype H3N2 was used. H3N2 is a subtype of influenza A virus that causes flu. Such viruses can infect birds and mammals and are increasingly abundant in seasonal influenza, which kills an estimated 6309 people in the United States each year, including pneumonia and influenza causes^21^. Based on previous studies, the averaged mass and the diameter of the H3N2 virus are 161 MDa^22^ and 100 nm^23^. Here we approximate the structure of the virus as a nanosphere with a core-shell structure of opposite charge distribution. The shell (90% of the total mass) contains lipid, neuraminidase (NA), hemagglutinin (HA), and M-protein. The core (10% of the total mass) includes RNA and RNP. The reduced mass (m*) of virus is thus 14.5 MDa. From the literature^24^, force with 400 pN applied on the AFM tip can fracture the lipid envelope. Since the radius of the tip was 30 nm^24^, the threshold stress to fracture the shell was 0.141 MPa (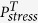). In order to calculate the threshold magnitude of the electric field to fracture H3N2 virus following equation (12), some important parameters such as *q*, *Q* and *ω*_*0*_ of the studied H3N2 virus has to be obtained by measuring the microwave absorption spectrum of viruses.

**Figure 3:**
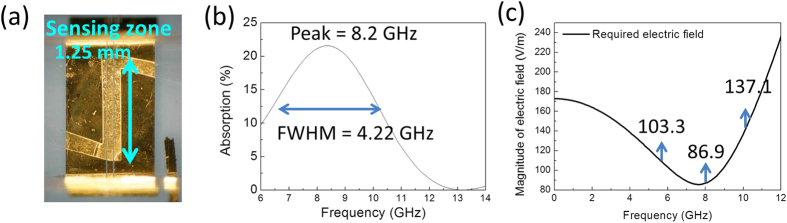
(**a**) Designed CPW circuit for microwave spectrum measured covered with a microfluidic channel. (**b**) Measured microwave absorption spectrum of H3N2 viruses. (**c**) Estimated threshold electric field magnitude to fracture the virus as a function of microwave frequency.

As shown in Fig. 3(a) we covered the structure of the coplanar waveguide (CPW) by the microfluidic channel with a 1.25 mm-long sensing zone (*L*) in order to measure the microwave absorption spectrum of viruses. This microwave microfluidic channel can provide a microwave bandwidth over 40 GHz. The measured results were summarized in Fig. 3(b). As the figure shows, the power absorption ratio (α) by the virus at the resonant frequency (8.2 GHz) was 21% and the *Q* was only 1.95 by measuring the full width at half maximum of the spectrum. Since the density of viruses (*N*) in the solution was 7.510^14 ^m^−3^, experimental absorption cross section of the virus at the resonant frequency can be calculated by the equation below:


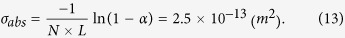


From equation (10), the theoretical absorption cross-section of the virus at the resonant frequency is:


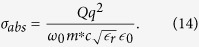


By setting 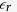
of the PBS at 8.2 GHz as 67.13^25^, 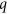 = 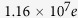 can be obtained by comparing equation (13) and equation (14).

So far, all parameters for estimating electric field threshold in equation (12) are obtained. By substituting threshold *P*_*stress*_* = *0.141 *MPa*, 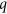 = 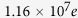, m* = 14.5 MDa, *Q* = 1.95 and *ω*_*0*_ = 2π × 8.22 GHz into equation (12), threshold magnitude of electric field to fracture virus at different frequencies of microwave can be calculated. The result is shown in Fig. 3(c). In order to compare with the following inactivation experiments, our estimated threshold magnitude of electric field at 6, 8 and 10 GHz are 103.3, 86.9 and 137.1 V/m, respectively. The minimum threshold occurs close to 8 GHz due to resonance, and sufficient internal stress to fracture virus can be expected to be more efficiently generated by weaker electric field.

Based on the IEEE Microwave Safety Standard, the spatial averaged value of the power density in air in open public space shall not exceed the equivalent power density of 100(*f*/3)^1/5^ W/m^2^ at frequencies between 3 and 96 GHz (*f* is in GHz)^26^. This corresponds to 115 W/m^2^ at 6 GHz, 122 W/m^2^ at 8 GHz, and 127 W/m^2^ at 10 GHz for averaged values of the power densities in air. Assuming all the microwave power in air 100% transmitted into a specimen, and by taking the dielectric constant of water 71.92 (6 GHz), 67.4 (8 GHz), and 63.04 (10 GHz)^25^ for calculation, this safety standard then corresponds to the average electric field magnitude of 101 V/m (6 GHz), 106 V/m (8 GHz), 110 V/m (10 GHz) inside the water-based specimens. It is interesting to notice that the required threshold electric field magnitudes at the resonant frequency (86.9 V/m) to fracture H3N2 viruses as shown in Fig. 3(c) are within the IEEE Microwave Safety Standard (106 V/m), indicating high SRET efficiency, even though the quality factor of the H3N2 virus is low.

## Results

### Virus Inactivation Experiments – Frequency dependency

**Figure 4:**
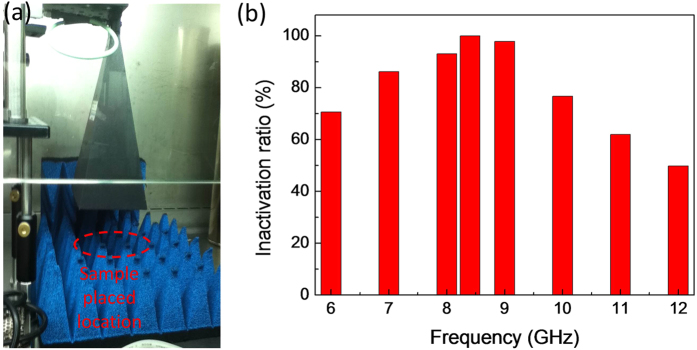
(**a**) Experimental setup for microwave illumination with different frequencies. (**b**) Inactivation ratio of H3N2 viruses after illuminating microwave with different frequencies.

To investigate the resonant effect, we first measured the residual viral infectivity of influenza A virus after illuminating microwave of different frequencies. As shown in Fig. 4(a), viral samples were placed below the horn antenna. The sponge under the sample was used to decrease the reflection of the microwave. To check the inactivation ratio, illuminated viruses were then analyzed by plaque assay to measure the residual infectivity of viruses. We compared the titer of illuminated viruses (*N*_*test*_) and the titer of unilluminated control sets (*N*_*control*_) to calculate the inactivation ratio (*1 − N*_*test*_*/N*_*control*_) at different frequencies between 6–12 GHz.

Based on Fig. 3(c), the field intensity threshold for inactivating H3N2 virus ranges between 86.9–236.3 V/m, which corresponds to 82.3–564 W/m^2^, for microwaves between 6 and 12 GHz. Since the aperture size of our horn antenna was 9.8 cm × 7.1 cm. The required threshold power input ranges from 0.57 W to 3.92 W for 6–12 GHz microwaves. We thus first applied 6.3 W (38 dBm) fixed microwave power, which is higher than all the threshold power input, into the horn antenna for the frequency dependency studies. After considering the transmission coefficient of our horn antenna, this experimental condition corresponded to 765 – 882 W/m^2^ average illuminated power density on the sample surface, corresponding to the field intensity inside the specimen of 260–296 V/m respectively. For 8–8.4 GHz microwave at the resonant frequency, the average illuminated power density was about 810 W/m^2^, equivalent to 273 V/m effective field intensity inside the sample. We thus expect to observe the inactivation effect throughout the studied spectral range. As been summarized in Fig. 4(b), a frequency dependent inactivation ratio can be observed in our experiments, with a peak located at the resonant frequency of the dipolar mode while higher than 50% inactivation ratio can be observed throughout the studied frequency range. At 8.4 GHz, the measured titer count was zero, indicating 100% inactivation ratio, which means that the remaining active viral concentration was smaller than the system sensitivity of 10 pfu/mL. This result indicates at least a three-order of magnitudes attenuation on the virus titer, when the microwave frequency was tuned to the dipolar mode resonant frequency with the electric field intensity 3 times higher than the threshold. The illuminated average power density was roughly 6.7 times higher than the IEEE safety standard for the 8–8.4 GHz cases. It is important to notice that the power density is proportional to the square of the field intensity.

### Virus Inactivation Experiments – Power density dependency

**Figure 5:**
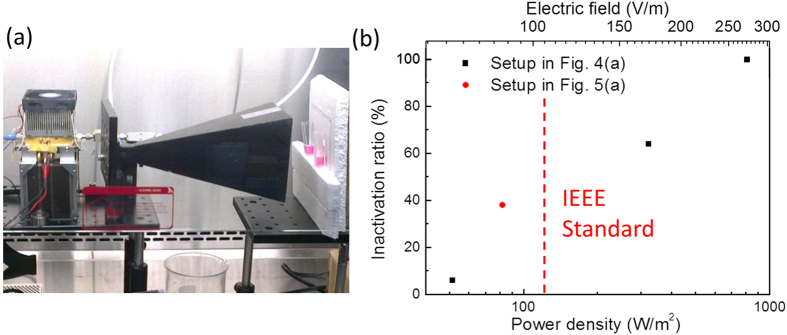
(**a**) Experimental setup for the red solid circle in Fig. 5(b). (b) Inactivation ratio for viruses illuminated by microwave with different power densities. The red solid circle was performed with the experimental setup shown in Fig. 5(a) and the black solid squares were performed with the experimental setup shown in Fig. 4(a).

To further investigate the efficiency of this SRET effect from microwave to virus and the threshold effect, we further measured the inactivation ratio of H3N2 virus with different power densities at the resonant frequency ~8 GHz of the confined acoustic dipolar mode. Our theoretical model predicted an inactivation threshold field intensity of 86.9 V/m, corresponding to an average microwave power density of 82.3 W/m^2^ in specimen. Since we assume all power can transmit from air to specimen, power density in air is also 82.3 W/m^2^, which is 1.48 times lower than the IEEE safety standard. Figure 5 (b) summarized the measured inactivation ratio for 4 different average microwave power densities of 820, 320, 82, and 51 W/m^2^ in air, corresponding to an effective field intensity inside samples of 274, 171, 87, and 68 V/m, respectively. It is noted that the experiment with 82 W/m^2^ in air was performed in a different experimental setup, as shown in Fig. 5(a). A significant threshold effect can be observed when the effective field intensity inside samples started to be on the order of or exceed the estimated threshold. A 38% inactivation ratio can be observed with a field intensity of 87 V/m, while the inactivation ratio dropped drastically to only negligible 6% with a slightly lower 68 V/m field intensity. With a 3 times higher field intensity than the threshold, the inactivation ratio saturated at a 100% value.

## Discussions

Compared with the simple theoretical model for threshold estimation as summarized in Fig. 3(c), our result agrees qualitatively and surprisingly quantitatively. First, in our experiments, we observed a strong resonant effect on the virus inactivation ratio at the dipolar oscillation frequency of 8.4 GHz, thus indicating that the observed virus inactivation after microwave illumination was due to the proposed SRET from microwave to virus through dipolar coupling. Second, at the resonant frequency, we do observe H3N2 virus inactivation by illuminating 82 W/m^2^ (lower than the IEEE safety standard in public space) 8 GHz microwaves on our viral solution, corresponding to an average 87 V/m electric field intensity inside the solution, confirming that our proposed simple model to estimate the field threshold (86.9 V/m) to structurally fracture the virus is quantitatively correct, especially combining the observed threshold effect as discussed above. With a low resonator quality factor (around and less than 2 for H3N2), we also observed virus inactivation in off-resonant frequencies (6-12GHz), following a trend predicted with our model. However for off-resonant frequencies, the simple harmonic oscillator model seems to always estimate a lower threshold in the Stoke-side (lower-frequency-side) of the resonant frequency than the anti-Stoke-side (high-frequency-side). For example, at DC (0 frequency) it still predicts a relatively low threshold field magnitude to fracture the virus. This is different from our observation. We observe that the anti-Stoke-side is with a better inactivation ratio than the Stoke-side, and the source for disagreement should be the over-simplification of the adopted model.

Our result regarding the efficient SRET to inactivate virus with a low microwave power has a profound meaning. As we introduced, in the past few decades, tremendous efforts have been made to kill airborne viruses such as SARS or influenza A, which have caused catastrophic illness worldwide. Active airborne viruses are always transported inside tiny water droplets, thus similar to our experimental condition. A strategy for airborne virus epidemic prevention in open public is thus highly desired. Our finding represents the first possible mechanism to inactivate airborne viruses without affecting the open public, since the required microwave power could be within the IEEE safety standard. Comparing this work with traditional microwave thermal inactivation, previous works^27^,^28^ used a microwaves oven with more than 100 W to heat the phage suspension. The inactivation ratio could reach almost 100% by increasing the temperature of the phage suspension to 80 °C. In our case, 100% inactivation were achieved with 6.3 W (38 dBm) as the input power into the horn antenna at 8.4 GHz. A power reduction by more than a factor of 15 is achieved. It is however not possible to directly compare the irradiating power density, since the irradiating area was not provided in previous literatures. Nevertheless our work still shows sharp contrast to current methodologies, including strong chemical inactivation, UV irradiation, and microwave thermal heating with 100 W microwave power^27^,^28^, which are not safe for the open public.

A previous study^29^ has shown that to inactivate human H3N2 viruses through thermal heating, the temperature need to be higher than 55 °C. Compared with the 82 W/m^2^ radiated microwave power density (0.63 W required power input) in our resonant inactivation case, the current microwave thermal heating method to inactivate virus usually requires more than 100 W microwave power at 2.45 GHz^27^,^28^, which is way beyond the safety standard, in order to raise the sample temperature to be higher than 60 °C for protein denature. It is known that the microwave thermal heating has a weak frequency dependency between 6–12 GHz, and this is not the case for our frequency dependent result as shown in Fig. 4(b). To confirm that our observation is not due to the microwave thermal heating effect, we had monitored the sample temperature change during the microwave illumination experiments with a radiated power density of 486 W/m^2^ at a frequency of 6 GHz by using an infrared thermal imaging camera with a temperature accuracy of 0.05 °C (CHCT, P384-20). The temperature rise after 15 minutes radiation was 7 °C, from 27.5 °C up to 34.5 °C. We thus exclude the possible contribution of microwave thermal heating effect to inactivation H3N2 viruses under our experimental condition.

**Figure 6:**
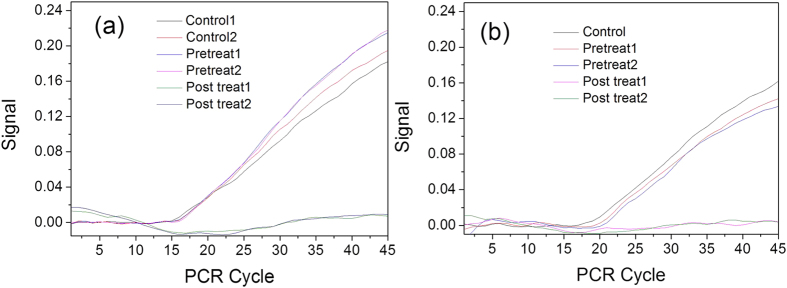
The RNA count for each amplification cycle of (**a**) H3N2 and (**b**) H1N1 viruses.

To double-confirm our proposed mechanism that the inactivation was through physically fracturing the structure of viruses, we established a fractured virus model by freezing the virus samples with liquid nitrogen and thawed immediately and repeated several times. We then preformed real-time RT-PCR (Reverse Transcription Polymerase Chain Reaction) experiment for RNA amplification in order to compare the results after microwave illumination with the established fractured-virus model. Without protein denature, the established fractured-virus model allowed the viral RNA content to be extracted after fracturing. We then performed the RT-PCR experiments to amplify the extracted viral RNA after fracturing either through the frozen fracturing model or after microwave resonant irradiation. Figure 6(a) summarized our results. The applied average microwave power density was 320 W/m^2^, the microwave frequency was tuned to the dipolar mode resonant frequency of 8.35 GHz, and the illumination time was 15 minutes. To avoid possible existing viral RNA in solution before the microwave illumination, we pretreated the virus samples by adding RNase to degrade the existing RNA outside the viral particles. As can be revealed in Fig. 6(a), the control fractured-virus model (shown as “control”) showed the same RNA amplification trend with excellent quantitative agreement with the RNase-pretreated samples after microwave resonant illumination (shown as “pretreat”). Obvious increase of copies in PCR can be observed after cycle 15. We have also performed two post-treat groups to double confirm the effect of the RNase. For post-treat groups, RNase was added right after the microwave resonant illumination. Even if the virus particles were fractured, the released RNA would be degraded by RNase, we did not expect to detect the viral RNA. As been also confirmed by our RT-PCR experiment as shown in Fig. 6(a), we were not able to detect the signal of RNA for the post-treat groups even after 45 cycles.

Since all the RNAs outside the virus surface in the solution were eliminated before the microwave illumination, for the pretreat samples the only way to detect the RNA signal after the illumination was to fracture the virus so that the RNA can be released. This result was also in good agreement on the amount of amplified RNA in the control cases. These facts confirmed that the inactivation of viruses by illuminating microwaves at the dipolar mode resonant frequency was through physically fracturing the lipid-envelope of the influenza virus without denaturing the viral RNA.

To show that the investigated SRET effect can be applied to deactivate viruses other than H3N2, we have also performed the real-time RT-PCR experiment on H1N1 virus. The result was shown in Fig. 6(b). The applied microwave frequency was 7 GHz, while the applied average microwave power density was 308 W/m^2^, corresponding to an effective electric field of 167 V/m inside the specimens. The rest of the experimental conditions were the same as that of Fig. 6(a). Similar results can be observed, indicating that the same SRET induced inactivation effect can also occurs in virus other than H3N2.

In summary, we investigated the structure resonance energy transfer from microwave to CAVs of H3N2 virus in water-based solution. The efficiency of such energy transfer was investigated through exploring the virus inactivation ratio. Based on the proposed damped mass-spring model and the experimentally measured microwave absorption cross-section of a single virus, threshold magnitude of electric field to fracture viruses at different illuminated frequencies can be estimated. After the illumination by the microwave, the plaque assay experiment indicated that the inactivation ratio reaches its maximum at the resonant frequency of the dipolar resonance. The real-time RT-PCR experiment double confirmed that the main inactivation mechanism was through physically fracturing viruses without degrading viral RNA genome. This work not only theoretically and experimentally demonstrates a new energy transfer mechanism between EM waves and viruses, but also indicates an efficient SRET effect. Our results have important implications for the interaction between microwaves and biological tissues, which is a highly concerned public issue. With an observed inactivation threshold with a microwave power density within the IEEE safety standard, the demonstrated SRET mechanism also provides a pathway toward establishing a new epidemic prevention strategy in open public for airborne viruses.

## Methods

### H3N2/H1N1 Sample Preparation

To prepare the H3N2 and H1N1 virus samples, Madin-Darby canine kidney (MDCK) cells were confluent grown in 75 cm^2^ flasks, inoculated by influenza A virus H3N2 (H090103, NTU Hospital) or H1N1 (NTUH135/2009, NTU Hospital) with multiplicity of infection (MOI) of 0.01 and incubated at 37 °C in a 5% CO_2_ incubator for 2–3 days. When the cytopathic effect (CPE) of the inoculated cells reached to 3 + (75%), we harvested the viral supernatant. The viruses were aliquot and stored at −80 °C for further use.

### Microwave Absorption Spectrum Measurement

The microwave absorption spectrum measurement was performed by combining the coplanar waveguide (CPW) circuit with a microfluidic channel. As shown in Fig. 3(a), we covered the structure of the CPW by the microfluidic channel with a 1.25 mm-long sensing zone (*L*). The gap between the signal electrode and the ground electrode of the CPW was 25 μm. In order to decrease microwave loss on the electrodes, the gold layer thickness of the electrode was 1.2 μm. On the surface of electrodes, we grew a thermal isolator layer of silicon dioxide on the sensing zone by Plasma Enhanced Chemical Vapor Deposition (PECVD) to lower the temperature rise of fluids due to microwave dielectric heating^30^. We then used a network analyzer (Anritsu, MS2028C) as the source to measure the absorption spectrum of H3N2 viruses from 6 GHz to 14 GHz. The virus particle density in the solution was 7.5 × 10^14^ m^−3^. The spectrum of the solution without viruses was first measured as a reference; solution with viruses was later measured. By removing the solution background, the microwave absorption spectrum of H3N2 viruses in solution was thus obtained (Fig. 3(b)) following a procedure similar to ref. 8.

### Microwave Illumination Measurement

In our experiments, we used two different microwave sources, depending on the utilized microwave power: a network analyzer (Anritsu, MS2028C) or an Yttrium iron garnet (YIG) oscillator. Then the microwave signal was amplified by a power amplifier (QPJ-06183640) and radiated from the horn antenna (ELECTRO-METRICS, EM-6969). The aperture size of the antenna was 9.8 cm × 7.1 cm. To avoid damage on oscillator and amplifier caused by back reflection, we added an isolator and a directional coupler. All the microwave systems were put in a P2 class flow hood. The antenna was directed toward the bottom (Fig. 4(a)) or the side (Fig. 5(a)) of flow hood and the microwave was normally incident on either microscope slides (Fig. 4(a)) or acrylic cuvettes (Fig. 5(a)) at a distance of 5 cm below the exit of horn antenna. To avoid large reflection from the metal hood surface, the microscope slide or cuvette was put in a plastic dish supported by a broad band pyramidal absorber. For each measurement, the viral solution (7.5 × 10^14^ m^−3^ particle density) under illumination was drop on the slide and covered with a cover glass or was contained inside the cuvettes. Under such an experimental geometry, we illuminated viruses for 15 minutes at various microwave frequencies or at various microwave powers. After illumination, we used buffer to wash-down and collect the viruses, which introduced a 10-fold dilution of virus concentration. Then the illuminated viral solutions, together with the control sets were sent for plaque assay.

### Quantitative Plaque Assay Analysis

To measure the activity of viruses, we employed a quantitative plaque assay. The MDCK cells used for plaque assay were grown in 6 wells plates by adding 3 mL of cells (2 × 10^5^/mL) to individual well. After confluent growth of MDCK cells in plates, the cells were successively washed with phosphate buffer saline (pH 7.2) and Eagles’ MEM with 2 μg/mL TPCK trypsin. After washed, 100 μL of the ten-fold serial dilution of viruses were added into each well. For better virus adsorption, the inoculated cells were incubated at 37 °C in a 5% CO_2_ incubator for one hour. After then, the virus inoculums were removed and the Eagles’ minimal essential medium with 2 μg/mL TPCK trypsin and 0.5% agarose was added. After the gel formation, the plates were put in a 5% CO_2_ incubator for at least 42–48 hrs. After incubation, the plates were fixed with 10% formalin for 1 hour. After pour off agarose, the fixed cells were stained with crystal violet for 15 min and washed with tap water. Below certain virus concentration, the plaques wouldn’t overlap each other seriously and can be counted unambiguously. Considering the corresponding dilution factor, the plaque forming unit per mL (pfu/mL) of the original virus can thus be quantified. The titer measurement will be performed three times on the same sample to reduce quantization error. Quantified by the plaque assay, the concentration of active viruses in our prepared viral solutions was around 10^7^/ml for H1N1 and H3N2.

### Real-time PCR Analysis

The sample RNA was extracted and amplified by the RT and quantitative real-time PCR (Primerdesign Precision OneStep™ qRT-PCR Mastermix) with primer AMF (sequence: 5′-GAGTCTTCTAACCGAGGTCGAAACGTA-3′), primer flu-AR (sequence: 5′-CAAAGCGTCTACGCTGCAGTCC-3′) and flu A (5′-FAM-tttgtgtttacgctcaccgt-TAMRA-3′) probe. For control group, RNase was added for 10 minutes digestion and stopped with RNase inhibitor. Then viruses were fractured artificially by freeze-thaw treatment. We thus were able to observe that the RNA signal of virus rose after 15 cycles of the amplification. For pre-treated groups, the RNase treatment were done and stopped before 8 GHz microwave illumination in order to make sure that RNA outside the viral envelope was eliminated in the first place. For the post-treat groups, RNase was added right after the illumination. If the virus particles were fractured, the released RNA would be degraded by RNase.

## Additional Information

**How to cite this article**: Yang, S.-C. *et al*. Efficient Structure Resonance Energy Transfer from Microwaves to Confined Acoustic Vibrations in Viruses. *Sci. Rep*. **5**, 18030; doi: 10.1038/srep18030 (2015).

## Supplementary Material

Supplementary Information

Supplementary Movie S1

Supplementary Movie S2
