# Supplementary material for: Efficient Structure Resonance Energy Transfer from Microwaves to Confined Acoustic Vibrations in Viruses
Source: Sci Rep. 2015 Dec 9;5:18030. doi: 10.1038/srep18030 (PMC4673452; doi:10.1038/srep18030)
Supplement: Supplementary Information [file srep18030-s1.pdf]

Supplementary Info

Efficient Structure Resonance Energy Transfer from Microwaves to Confined Acoustic Vibrations in Viruses

Szu-Chi Yang[1], Huan-Chun Lin[2], Tzu-Ming Liu[3], Jen-Tang Lu[1], Wan-Ting Hung[4], Yu-Ru Huang[1], Yi-Chun Tsai[1], Chuan-Liang Kao[2], Shih-Yuan Chen[4], and Chi-Kuang Sun[1,5a)]

[1] *Department of Electrical Engineering and Graduate Institute of Photonics and Optoelectronics, National Taiwan University, Taipei 10617, Taiwan*

[2] *Department of Clinical Laboratory Sciences and Medical Biotechnology, College of Medicine, National Taiwan University, Taipei 10617, Taiwan*

[3] *Institute of Biomedical Engineering, National Taiwan University, Taipei 10617, Taiwan*

[4] *Department of Electrical Engineering and Graduate Institute of Communication Engineering, National Taiwan University, Taipei 10617, Taiwan*

[5]*Molecular Imaging Center and Graduate Institute of Biomedical Electronics and Bioinformatics, National Taiwan University, Taipei, 10617, Taiwan*

Breathing Mode and Dipolar Mode of Virus

The eigenvalue equation of the spheroidal modes of a homogeneous sphere (Supplementary Figure 1) can be expressed as:

$$4\left[\eta^2+(\ell-1)(\ell+2)\left(\frac{\eta j_{\ell+1}(\eta)}{j_\ell(\eta)}-\ell-1\right)\right]\frac{\xi j_{\ell+1}(\xi)}{j_\ell(\xi)}-\eta^4$$

$$+2(\ell-1)(2\ell+1)\eta^2+2[\eta^2-2\ell(\ell-1)(\ell+2)]\frac{\eta j_{\ell+1}(\eta)}{j_\ell(\eta)}=0 \text{ for } \ell\geq 1, \quad (1)$$

and

$$\frac{4{c_t}^2 j_1(\xi)}{{c_l}^2\xi}-j_0(\xi) \text{ for } \ell=0 \quad (2)$$

where $\xi=\frac{\omega R}{c_l}$, $\eta=\frac{\omega R}{c_t}$, j_ℓ is the spherical Bessel function of the first kind, ω and ℓ are the angular frequency and angular momentum of the vibrational mode, R is radius

of the nano-sphere, c_l and c_t are longitudinal and transverse sound velocities respectively. Depending on different values of ℓ, different modes of a sphere can be derived. Supplementary Movie 1 shows animations of the total displacement field of the breathing mode ($\ell = 0$) and Supplementary Movie 2 shows the displacement field in z direction of the dipolar mode ($\ell = 1$) on the x-z plane of the sphere (coordinate is defined in Supplementary Figure 1). One can observe that the displacement of the breathing mode is radial symmetric and this mode can be detected in the most of light scattering experiments. This well-known breathing mode is NOT the mode responsible for efficient energy transfer between microwaves and viruses. On the other hand, the dipolar mode shown in Supplementary Movie 2 is the mode responsible for the efficient energy transfer between microwaves and viruses. The displacement of the dipolar mode is along the direction of the electric field and it is necessary to have the permanent charge separation in the sphere for exciting the dipolar mode. In our experiment, the energy of the electromagnetic waves is directly transferred to the dipolar mode of spherical viruses when on resonance.

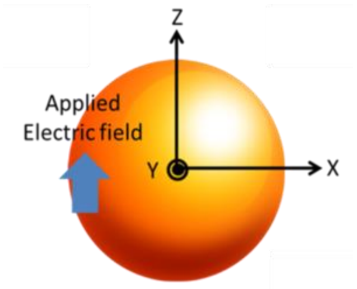

Supplementary Figure 1. Schematic showing a homogeneous sphere and applied electric field.

Supplementary Movie 1. Animation showing the total displacement field of the breathing mode ($\ell = 0$).

Supplementary Movie 2. Animation showing the total displacement field in z direction of the dipolar mode ($\ell = 1$) on the x-z plane of the sphere (coordinate is defined in Supplementary Figure 1). This is the mode responsible for the efficient energy transfer from microwave to a spherical virus like H3N2.
